# A computational study of cooperative binding to multiple SARS-CoV-2 proteins

**DOI:** 10.1038/s41598-021-95826-6

**Published:** 2021-08-11

**Authors:** Jianing Li, Kyle T. McKay, Jacob M. Remington, Severin T. Schneebeli

**Affiliations:** Department of Chemistry, University of Vermont, Burlington, VT 05405 USA

**Keywords:** Virtual screening, Computational chemistry

## Abstract

Structure-based drug design targeting the SARS-CoV-2 virus has been greatly facilitated by available virus-related protein structures. However, there is an urgent need for effective, safe small-molecule drugs to control the spread of the virus and variants. While many efforts are devoted to searching for compounds that selectively target individual proteins, we investigated the potential interactions between eight proteins related to SARS-CoV-2 and more than 600 compounds from a traditional Chinese medicine which has proven effective at treating the viral infection. Our original ensemble docking and cooperative docking approaches, followed by a total of over 16-micorsecond molecular simulations, have identified at least 9 compounds that may generally bind to key SARS-CoV-2 proteins. Further, we found evidence that some of these compounds can simultaneously bind to the same target, potentially leading to cooperative inhibition to SARS-CoV-2 proteins like the Spike protein and the RNA-dependent RNA polymerase. These results not only present a useful computational methodology to systematically assess the anti-viral potential of small molecules, but also point out a new avenue to seek cooperative compounds toward cocktail therapeutics to target more SARS-CoV-2-related proteins.

## Introduction

Despite the availability of vaccines, there is still an urgent need for small molecules that are effective against SARS-CoV-2, the virus which causes the COVID-19 disease in the current pandemic. Over 300 drugs are being studied as potential repurposed candidates, but to date only remdesivir^1^ has been approved to treat COVID-19 in the US with an Emergency Use Authorization (EUA). In fact, we still must discover new small molecules for use as safe, oral antivirals to treat patients early in the course of infection, which will be key to control the spread of the virus. Also, there is an urgent need for effective treatments or even cures for patients with severe symptoms, as well as to prepare us for emerging variants of SARS-CoV-2. Thanks to a timely response from the global research community^2^, over 1000 virus-related protein structures have been deposited to the Protein Data Bank (PDB) since the start of the pandemic. Particularly, the RNA genome of SARS-CoV-2 and sequences of the encoded 29 proteins have been revealed^3,4^, and the structures of more than nine unique viral proteins are available, all of which greatly facilitate computational efforts to search for compounds as potential treatments of COVID-19. In this work, we present a systems computational study that targets multiple SARS-CoV-2 proteins that span its genome and examine the potential of natural compounds for cooperative inhibition, aiming to inspire a new avenue towards finding compounds with cooperative effects as potential therapeutic agents.

So far, several directions of structure-based drug design (SBDD)—such as inhibitions of viral entry, assembly, replication, etc.—have been pursued, mainly targeting a few structural and non-structural proteins of SARS-CoV-2. Among the four structural proteins, the Spike protein (S protein), which is crucial for host cell recognition and entry, is a primary target^5,6^. Compounds that block the interactions between the S protein and the angiotensin converting enzyme 2 (ACE2) are believed to prevent viral entry and subsequent infection^7–10^. Besides the S protein, two key proteases, the main protease (3CL^pro^ or M^pro^) and the papain-like protease (PL^pro^), which are encoded as part of non-structural proteins 5 and 3 (nsp5 and nsp3, respectively), are essential for the replication and assembly of SARS-CoV-2^11–13^. Inhibition of these two proteases has also become the focus of many research programs^14–16^. Finally, the RNA-dependent RNA polymerase (RdRp) encoded as nsp12 is a component necessary for viral replication and transcription and is considered another emerging target^17,18^. In addition to those named here, there is a fast-growing list of potential targets for SBDD^3,19–24^.

Despite the expanding breadth of targets, current computational studies almost exclusively focus on similar methodologies—the development of specific and highly selective compounds to inhibit individual proteins (the “one compound, one target” strategy). These methodologies are not without their challenges^14,25,26^. In particular, it remains a possibility that small molecules interact with multiple protein targets, and a protein target may allow cooperative or allosteric binding of the same or different molecules. Thus, conventional molecular modeling approaches like docking and virtual screening should be adapted to allow for such investigations. Ensemble docking, using representatives of protein conformational ensembles rather than a single structures, can be more accurate to identify potent compounds^27,28^ but is often computationally demanding. Therefore, we have designed a multi-step method to prescreen the compounds with conventional docking and use ensemble docking for selection, followed by a series of custom analytical steps, simulations, and cooperative docking (see Methods and models). Our methodology incorporates a carefully designed clustering algorithm and workflow, which is likely to balance computational accuracy and efficiency. In this work, we, for the first time, utilized this method in the spirit of systems biology, to extensively examine interactions between eight SARS-CoV-2-related proteins (Figure S1 and Table S1) and natural compounds to show the potential cooperative effects of small molecules against SARS-CoV-2.

We included six SARS-CoV-2 proteins and two ACE2 proteins (human and cat) in our receptor set, with small molecules (structures available for download in the SI) from 20 herbal ingredients in the *Qingfei Paidu* decoction (QPD)—a traditional Chinese medicine which underwent clinical trials in 2020–2021^29,30^. In a clinical trial with near 9000 patients hospitalized in China during the period from January to May 2020, the QPD treatment was found to reduce the COVID-19-related mortality significantly from 4.8 to 1.2%^30^. Despite such effectiveness, bioactive molecules in the 20 herbal ingredients of QPD and their mechanism of action remain largely unknown. Also, as QPD is administrated in clinical practice as a mixture of many bioactive compounds from the herbs, a systematic study is required to understand the individual and joint interactions between these compounds and the SARS-CoV-2 proteins. To fulfil this need, we have developed our computational methodology which is comprised of ensemble docking, cooperative docking, and extensive molecular dynamics (MD) simulations for the systems chemistry investigation. Through literature search, we have identified more than 600 flavonoids, triterpenes, polysaccharides, and other bioactive compounds which are considered most medicinally relevant in this work (see the selection criteria in Methods and Models). From this study, we have identified several compounds which potentially inhibit multiple SARS-CoV-2 proteins; compounds from the same or different herbs may have synergy to enhance binding to the viral proteins. These findings shed light on new directions of COVID-19 treatments.

## Results

### Flavonoid glycosides displayed medium to strong interactions with general SARS-CoV-2 proteins

**Figure 1:**
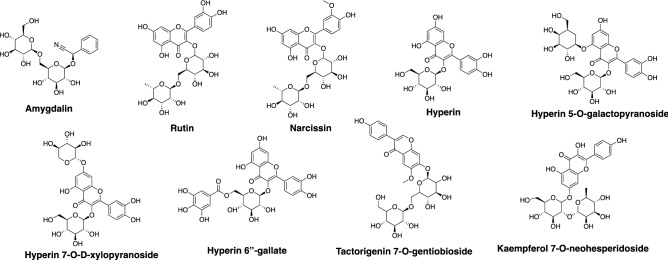
Two-dimensional structures of nine compounds suggested by ensemble docking to interact with multiple SARS-CoV-2 proteins and human/cat ACE2. Among them are eight flavonoids including Hyperin and three glycosylated derivatives as well as Amygdalin, a cyanogenic glycoside. These chemical structures were prepared in *ChemDraw (version 20.0)*.

We have identified at least nine compounds that broadly interact with the six SARS-CoV-2 proteins as well as the ACE2 enzymes, with affinities predicted mostly between − 5 and − 15 kcal/mol (Fig. 1 and Table 1). A typical example is rutin (C_27_H_30_O_16_), which is a flavonoid glycoside common in several ingredients of QPD such as those related to citrus fruits (*fructus aurantii immaturus* and *citri reticulatae pericarpium*, or known as Zhi Shi and Chen Pi in Chinese, respectively), as well as the Chinese thorowax root (known as Chai Hu). Despite debate about its antiviral effects^31,32^, rutin was suggested to block the active site of SARS-CoV-2 3CL^pro^ in recent computational studies^33–35^. While our results from ensemble docking are consistent with prior research in rutin binding to the 3CL^pro^ active site^34^, we revealed the interactions of rutin with other SARS-CoV-2 structural and non-structural proteins (Figure S1). Rutin can bind to the S protein’s receptor binding domain (RBD) and ACE2 (Glide XP score from − 5 to − 7 kcal/mol) and interact with residues at their recognition interfaces^7^, such as L455, F456, A475, G476, F486, N487, Y489, and F490 of the viral S protein; residues 19–45, 82–83, 330, 353–357 of the human/cat ACE2. Further, we also found stable binding (Glide XP score less than − 7 kcal/mol) of rutin to the active sites of PL^pro^ (rutin centroid distance to the S atom of the catalytic C111 is 10.7 ± 0.9 Å) and 3CL^pro^ (between the catalytic dyad of H41 and C145; rutin centroid distance to the S atom of C145 is 9.2 ± 0.4 Å), as well as the RNA binding site of RdRp (Y546, K593, S814, E854, R858, S861, D865, and Q932). The close contact to the catalytic cysteine or occupation of the active site suggests the potential for rutin to be an effective inhibitor to these viral enzymes. Similar to rutin, we also identified other flavonoid glycosides like hyperin and its derivatives which displayed medium to strong affinity to the six SARS-CoV-2 proteins. More than one stable binding mode (less than − 7 kcal/mol) was often captured in our ensemble docking poses. Notably, prior computational studies that screened various databases^13,24,36^ also identified a number of flavonoid glycosides as potential inhibitors to 3CL^pro^, PL^pro^ and RdRp, which strongly supports our observations in this work.

**Table 1:** Compounds suggested by ensemble docking to interact with multiple SARS-CoV-2 proteins and human/cat ACE2, as well as their best Glide XP docking scores (kcal/mol) from ensemble docking. The PDBID of each protein model was provided in parenthesis in the table header.

Compound	MW (g/mol)	S protein (7C8D)	Npro (6WJI)	3CL^pro^ (7JYC)	PL^pro^ (6WX4)	RdRp (7BV1)	NSP3 (5RSO)	cACE2 (7C8D)	hACE2 (6VW1)
Amygdalin	457	− 5.5	− 10.2	− 8.4	− 6.9	− 7.5	− 9.8	− 5.7	− 5.2
Rutin	611	− 7.2	− 8.0	− 12.2	− 7.8	− 9.3	− 10.5	− 7.3	− 5.1
Narcissin	625	− 4.8	− 8.8	− 11.2	− 6.6	− 8.0	− 9.3	− 5.9	− 6.1
Hyperin	464	− 7.1	− 10.2	− 10.4	− 7.1	− 7.7	− 10.5	− 6.5	− 4.3
Hyperin 5-O-galactopyranoside	627	− 7.6	− 8.7	− 11.4	− 9.0	− 10.3	− 10.5	− 7.0	− 6.0
Hyperin 7-O-D-xylopyranoside	597	− 6.2	− 11.4	− 10.6	− 6.1	− 9.5	− 12.3	− 6.3	− 6.2
Hyperin 6″-gallate	617	− 5.1	− 12.4	− 9.8	− 6.0	− 9.6	− 10.3	− 7.2	− 6.1
Tectorigenin 7-O-gentiobioside	625	− 6.2	− 14.1	− 11.4	− 6.5	− 10.0	− 9.9	− 6.3	− 5.5
Kaempferol 7-O-neohesperidoside	595	− 8.0	− 8.2	− 10.5	− 6.8	− 8.3	− 9.2	− 5.1	− 4.7

**Figure 2:**
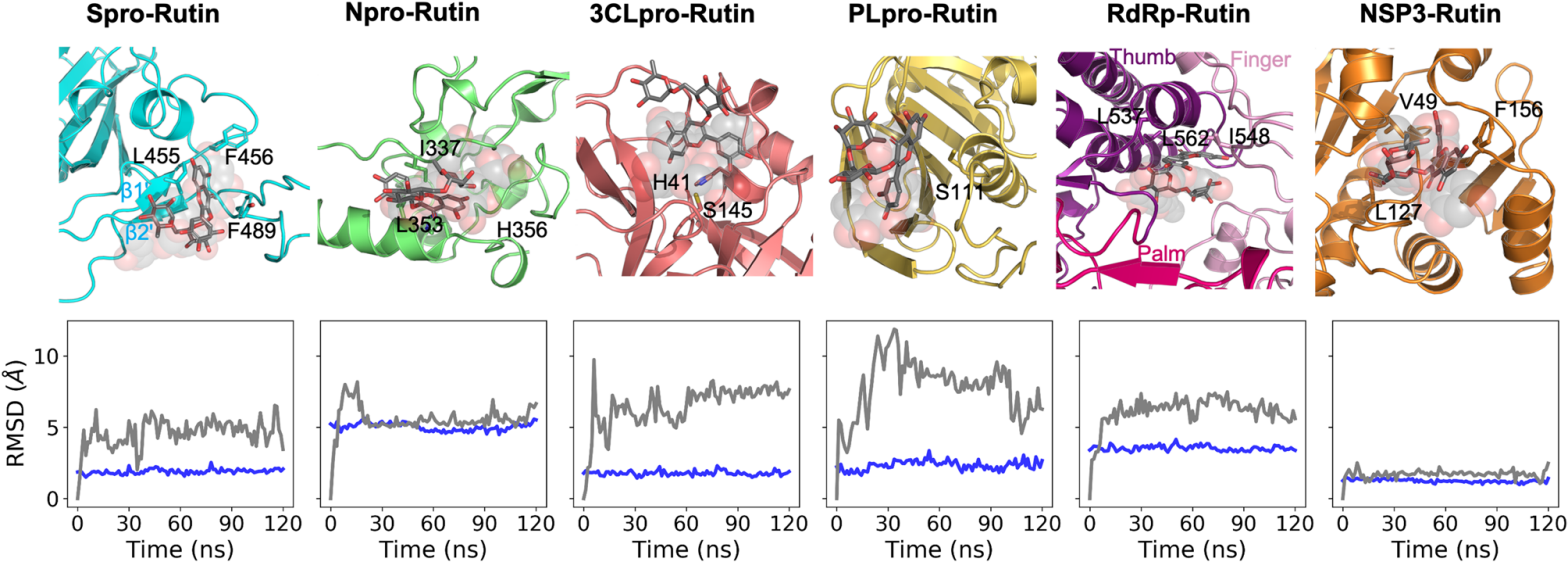
Cartoon illustration of rutin interactions with key SARS-CoV-2 proteins and the time evolution of RMSDs with protein alignment (blue plot: protein Ca atoms; grey plot: rutin heavy atoms). The RMSDs were calculated using the initial docking models as references. In the cartoon, the initial ligand positions (from docking) are represented as transparent spheres, and the final ligand positions (the final snapshot of the MD simulation) are shown as sticks. As the initial and final ligand positions largely overlap, it is shown that rutin stayed bound to the viral proteins through the simulations. The cartoon illustration was prepared in *Pymol (version 2.3.4)*.

To confirm the stability of the rutin-bound complexes, we performed 120-ns MD simulations of the best pose of each cluster from ensemble docking in solution, which helped us assess the stability of the ligand–protein complexes. The rutin molecules stayed bound to the proteins throughout most simulations (Table S4). With protein alignment, our ligand root mean square deviation (RMSD) mainly varied in the range between 1.6 and 9.7 Å (Fig. 2) for all complexes, comparable with previous simulations^34^ of rutin complexed with 3CL^pro^ (RMSD 4–6 Å). In particular, we observed that rutin was tightly bound in the macrodomain of nsp3, which has a well-defined pocket (Glide XP score of − 10.5 kcal/mol, ligand RMSD 1.6 ± 0.3 Å), but was much more dynamic when bound to other proteins with open or wide binding sites such as the S protein RBD and RdRp (ligand RMSDs of 4.7 ± 0.9 and 5.9 ± 0.5 Å, respectively). For example, key residues in the S protein RBD for ACE2 binding mainly involve the β1′ and β2′ regions^37^, which are quite extended (greater than 30 Å in length) with a large surface area (2456 Å^2^, calculated with the PDB structure 7C8D). In both ensemble docking and MD simulations, rutin has been shown to form a wide range of interactions with hydrophobic patches on the S protein RBD surface, including residues L455, F456, and F489 (Fig. 2). Similarly, various binding poses of rutin in the large RNA-binding site of RdRp (approximately 25 Å in width), first suggested by ensemble docking, remained stable during our simulations, mostly in contact to the thumb^38,39^ region of RdRp (Fig. 2). With comparable ligand stability to similar simulation studies ranging from 50 to 400 ns^9,12,13,34,40^, our simulations suggested that a number of small compounds from the QPD ingredients can bind to key SARS-CoV-2 proteins with medium to strong affinity. While our results identified individual compounds as “good binders” to these proteins, we also endeavored to address the question whether simultaneous binding of similar or different compounds can further enhance the binding strength.

### Structurally similar compounds binding to the S protein

We discovered from cooperative docking and MD simulations that a number of flavonoid glycosides (like rutin, narcissin, and chrysin 7-o-beta-gentiobioside, etc.) can simultaneously bind to the same viral protein. Generally, flavonoids share a 15-carbon skeleton comprised of two phenyl rings (namely A and B) and a heterocyclic C ring^41^ and readily form π–π-stacking with other flavonoids or similar compounds bound to a protein target. This has been observed before in a co-crystalized structure^42^. In addition, a distal sugar moiety may also allow hydrogen bonding to the protein, further stabilizing the complex. Considering that the S protein exists as a homotrimeric complex^43^, we notably predict simultaneous binding at even a single monomer. Using the S protein RBD with several flavonoid glycosides from our ligand set, we gained proof of concept that multiple compound binding may enhance the inhibition of the target viral protein.

**Figure 3:**
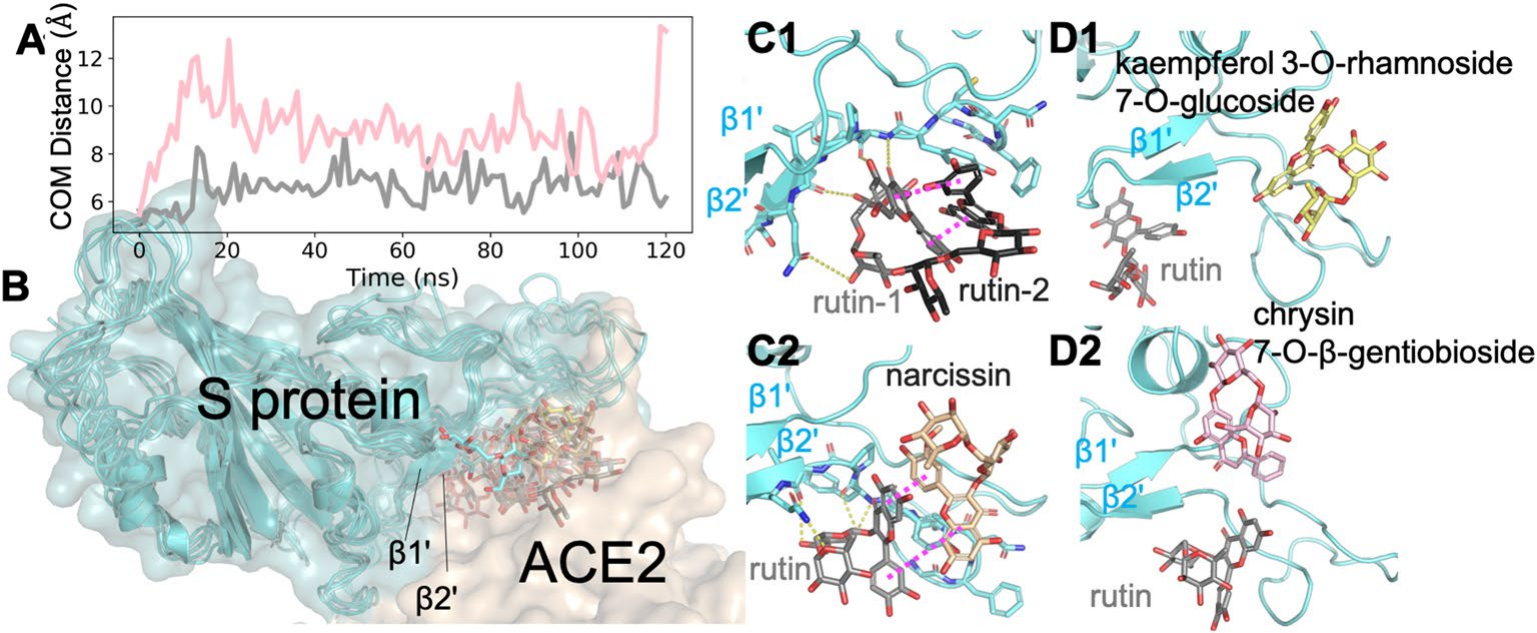
Cooperative and additive binding of two flavonoid glycosides to the S protein RBD. (**A**) Time evolution of the center of mass (COM) distances (grey plot: rutin-rutin; pink plot: rutin-chrysin 7-O-β-gentiobioside). (**B**) Cartoon illustration of typical flavonoid glycosides from QPD ingredients binding to the S protein-ACE2 interface, especially involving the β1′ and β2′ region (PDBID: 7C8D). (**C**, **D**) Final snapshots of MD simulations of two flavonoid glycosides binding to the S protein. Hydrogen bonds were shown in yellow dash; and π–π-stacking was shown with magenta dash. The cartoon illustration was prepared in *Pymol (version 2.3.4)*.

Our cooperative docking approach started by selecting a pose for a single ligand from ensemble docking. Next, the second ligand was docked to the same binding site (containing the first ligand), which generated initial models for subsequent MD simulations of the complex with two ligands. Notably, the assignment of the first and second ligands can affect the complex stability seen in subsequent MD simulations (see the Discussion section). For proof of concept, we only focused on selected compounds (i.e. Glide XP score less than -10 kcal/mol) and rutin in this work. For example, we have docked six compounds (Table S5) to the rutin-bound S protein model. These compounds displayed stronger binding (with docking scores lowered by 0.5–1.8 kcal/mol) as the second ligands than as the first ligands, which is indicative of cooperative binding. As expected, most of the secondary ligands were predicted to form π–π-stacking interactions with rutin. In the subsequent MD simulations, we found that all the compounds remained bound to the S protein (Fig. 3), and such stacking interactions were stable in four simulations. First, the complex with two rutin molecules was highly stable, while the rutin dimer remained bound to the β1′ and β2′ region. Particularly, the π–π-stacking between the rutin molecules was enhanced during sampling in the MD simulations (centroid distance of 6.7 ± 0.7 Å), leading to the packing of A/C ring from one compound and the B ring from another (Fig. 2C). The glucose and rhamnose sugar groups stretched out to interact with the side chain of Q493 and the backbone of residues 490–492. As a result of these interactions, the structural fluctuations of the protein (RMSD from 2.0 to 1.7 Å) and the first rutin (RMSD from 4.7 to 4.5 Å) were slightly reduced, compared with the simulation with one rutin bound (Table S4). Further, similar enhanced π–π-stacking interactions were observed in narcissin-rutin, coumarin glycoside-rutin, and tectorigenin 7-O-gentiobioside-rutin in complex with the S protein RBD, indicated by the centroid distance of the two ligands below 8.0 Å (Table S5). Thus, adding another flavonoid glycoside may increase the stability of the complex. Likely, these results imply potential synergy between the active compounds in QPD, regarding the same compounds (like rutin), compounds from the same herb (like rutin and narcissin in the Torowax root), or even compounds from different herbs (like rutin in the Torowax root and tectorigenin 7-O-xylosylglucoside in Belamcanda Sinensis). However, for the other pairs of compounds, the complex conformations from docking were less stable. While both ligands stayed at the S protein interface with ACE2, their centroid distances over 8.0 Å indicated little to no stacking between the two compounds (Fig. 3A, D), such as chrysin 7-O-β-gentiobioside-rutin (centroid distance of 8.8 ± 1.8 Å) and kaempferol 3-O-neohesperidoside-rutin (centroid distance of 20.0 ± 1.9 Å). In these cases, although the first ligand did not enhance the binding of the second ligand, it may still add inhibition of the S protein binding to ACE2 in comparison to only the first ligand.

In general, the above-described patterns of multiple compound binding—involving both addition and synergy—to the S protein were also observed in flavonoid glycoside binding to viral enzymes like 3CLpro, PLpro, and RdRp, as well as to human/cat ACE2 (Table S5). These findings confirm the possibility that cooperative compounds may target multiple viral proteins and thus likely act via various mechanisms in the life cycle of SARS-CoV-2.

### Structurally distinct compounds binding to the RNA polymerase

**Figure 4:**
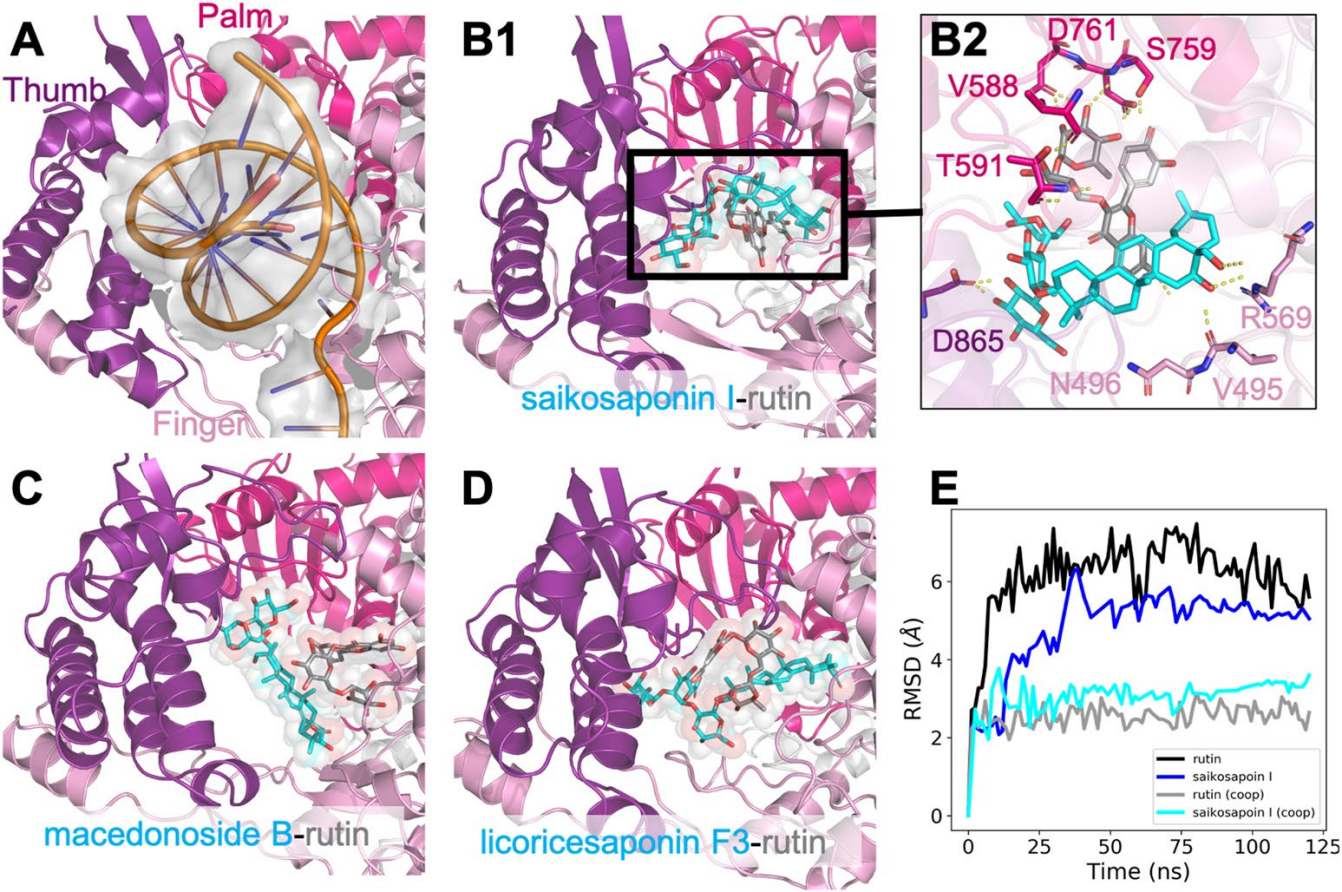
Cooperative binding of rutin and triterpenes at the RNA-binding site of RdRp. (**A**) Cartoon illustration of the RNA-binding site surrounded by the so-called Thumb, Palm, and Finger regions (PDBID: 7BV2). (**B**–**D**) Final snapshots of MD simulations of rutin and a triterpene compound binding to RdRp. (**E**) Time evolution of ligand RMSD of rutin or saikosaponin I-bound simulations as well as the saikosaponin I-rutin RdRp complex simulations. It is shown that the cooperative binding reduces the ligand flexibility at the wide RNA-binding site of RdRp. The cartoon illustration was prepared in *Pymol (version 2.3.4)*.

Different from the S protein, we found cooperative binding of two different types of compounds (like triterpenes and flavonoids) at the large RNA-binding site of RdRp. In our simulation of saikosaponin I and rutin bound to RdRp, there was clear hydrophobic interactions between the C30 skeleton of saikosaponin I with the A/C ring in rutin, as well as the sugar moieties forming hydrogen bonds with the RNA-binding site of the protein (Fig. 4B2). According to our simulations, rutin or saikosaponin I was relatively stable at the same site alone, indicated by the average ligand RMSD near 5 Å. However, while both molecules were bound to RdRp (Fig. 4E), the binding stability was greatly increased as shown by the low RMSDs (saikosaponin I, 3.4 ± 0.1 Å and rutin 2.6 ± 0.2 Å), and they remained stacked for 120 ns throughout the simulations (centroid distance of 5.8 ± 0.2 Å). In addition to saikosaponin I from Chinese thorowax root, we also simulated two other triterpenes from the licorice root, macedonoside B and licoricesaponin F3 (Fig. 4), which were also found to form strong stacking with rutin at the RNA-binding site of RdRp. Such interactions may involve tighter binding of the small compounds to RdRp, which likely block RNA binding to the polymerase.

In addition, we observed cooperative binding of two flavonoids (like rutin-rutin and rutin-tectorigenin 7-O-xylosylglucoside) at the RNA-binding site of RdRp (Table S5). Taken all together, as a potential QPD molecular mechanism to treat COVID-19, active compounds from different herbs may simultaneously bind the viral proteins and generate additional or synergistic inhibitory effects.

## Discussion

From the drug discovery perspective, some of the SARS-CoV-2 proteins can be difficult targets for structure-based design, given their less well-defined (like in the S protein) or generally very large binding pockets (like in the RdRp). Thus, it can be a challenge to identify specific and selective compounds. From ensemble docking and molecular simulations, we identified several compounds contained in QPD that potentially bind to the viral proteins, but most of them displayed only medium affinity with multiple possible binding poses, which renders these compounds less attractive as potential drug candidates. However, with ensemble/cooperative docking combined with extensive MD simulations, we have examined the possibility of multi-compound interactions at the same binding site. We found that binding of one compound (i.e. rutin) from QPD ingredients to a viral protein can favorably influence the binding of another compounds (i.e. another flavonoid or a triterpene) from the same or different ingredients, mostly likely through π–π-stacking interactions. Cooperativity of ligand binding through direct interaction between stacked molecules, often referred to as heterotropic cooperativity, has been long known in model proteins like P450 3A4^42,44^. Interestingly, the stacking stability can change with different assignments of the first and second ligands (or the docking order of cooperative compounds). Regarding the cooperativity of rutin and lucenin3 when binding to 3CL^pro^ (Table S5 and Figure S3), the stacking between lucenin3 and rutin were much stronger (centroid distance of 7.6 ± 0.3 Å), with lucenin3 directly bound to 3CL^pro^ as the first ligand (RMSD of 1.2 ± 0.2 Å). However, with rutin as the first ligand (RMSD of 6.2 ± 0.3 Å), the complex appeared less stable and the stacking was much weaker (centroid distance of 10.6 ± 0.3 Å). The complex that is more thermodynamically stable will be likely dominant, and further theoretical and experimental studies may be needed in the future to fully explore this phenomenon. In addition to cooperative binding, we also found different compounds that may bind to the large interface of the S protein and ACE2, which generates a joint effect stronger than a single compound. Overall, our work shows that QPD provides a rich source of active small molecules that may synergistically inhibit the key players in the processes of SARS-CoV-2 entry, assembly, and replication.

**Figure 5:**
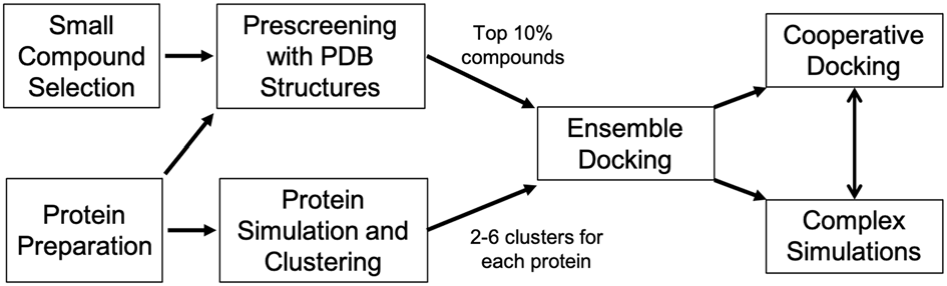
Cartoon illustration of our workflow. Prescreening used the Virtual Screening Wizard and ensemble/cooperative docking was performed with Glide with the Glide XP scoring function.

Although we only gained proof of concept for possible synergy among selected compounds in a 1:1 stoichiometry, more complex binding (e.g. more than two different molecules, or different stoichiometry) is possible. Our ensemble and cooperative docking approach (Fig. 5) can be utilized to further explore these possibilities at an affordable computational cost. Moreover, although only selected SARS-CoV-2 proteins with crystal structures were studied in this work, the methodology presented here opens the door to more comprehensive studies to cover (i) other viral and even human protein targets (with experimental structures or theoretical models), and/or (ii) more small molecules (e.g. from databases). Notably, our results suggested only 12 of 20 QPD ingredients that may be directly associated with SARS-CoV-2 interactions. Some other herbal ingredients may be involved to regulate the human immune system, and interact with human therapeutic targets. It is viable to establish more comprehensive studies on the methodology in this work, which can complement current efforts in computer-aided discovery for COVID-19 treatments.

## Conclusions

We have systematically modeled the interactions between eight related proteins and more than 600 small molecules from herbal ingredients of a Chinese medicine against SARS-CoV-2. Our results suggest that several natural compounds may be able to inhibit the viral proteins, and thus key processes in the viral life cycle. We also found compelling evidence that selected compounds may simultaneously bind to the same viral protein as the Spike protein and the RNA polymerase, leading to synergistic effects in blocking the protein targets. While the synergy of identified compounds will be validated in the future experimentally, we provide a novel computational protocol to discover synergistic agents to target large protein binding sites, as a valuable tool to discover new COVID treatments.

## Methods and models

### Small-molecule ligand selection and preparation

Distinct from prior studies to screen compounds from databases^13^, we collected a set of 625 small-molecules (compound names and 3D models provided in the SI for download) from a thorough literature search from recent analytical studies of bioactive compounds of 20 herbs in QPD (Table S1). After referring to records in PubChem^45^, volatile or toxic compounds were excluded, so that we could focus on the chemically relevant ingredients. Notably, QPD is prepared by boiling the dried herbal components in the gypsum aqueous solution for 20–30 min, and the small, volatile compounds are likely lost during the drying and/or boiling processes. Non-volatile compounds are more likely to be dominant in the active ingredients of QPD. The 3D models of these compounds were either built in *Maestro* (Schrödinger *Inc.*) or downloaded from PubChem, followed by structure cleanup and prediction of the favorable tautomeric states in the *Epik* program^46^. The qualitative assessment of absorption, deposition, metabolism, excretion and toxicity (ADMET) profile of selected hits were predicted computationally by using the SwissADME server^47^ (Table S6).

### Ensemble and cooperative docking approaches

We have created an original approach for ensemble docking, which efficiently assesses the potential interactions between a large number of compounds (ligands) and conformations of multiple protein targets (receptors). The work flow of this approach, illustrated in Fig. 5, was designed to balance the computational efficiency and accuracy in a systems chemistry study. The eight PDB structures (Table 1 and Table S1) of protein receptors (six SARS-CoV-2 proteins, the human ACE2, and the cat ACE2) were prepared by the Protein Preparation Wizard in *Maestro* (Schrödinger, Inc.), to assign protonation states and to fill incomplete side chains and loop gaps^48,49^.

After preparation of the small-molecule compounds and the protein receptors, our approach starts with virtual screening using one model for each receptor. Notably, most selected PDB structures in this work have co-crystalized ligands and known binding interfaces, so that we could readily define the ligand-binding sites. The docked ligand is confined to the enclosing box centered at the binding-site center (10 Å for the inner box; 10 or 15 Å for the outer box). While all the 625 compounds were prescreened using the Virtual Screening Wizard (Schrödinger *Inc.*), the top 10% with the best Glide XP scores were then promoted to ensemble docking. To do so, we could filter the compounds that are too small or too bulky for binding, and allow only the most relevant compounds for the costly ensemble docking and subsequent stages in our workflow. Visualization of the docking results was carried out in *Maestro (version 2020-2)* and *Pymol* (*version 2.3.4*).

In parallel to prescreening, the receptor conformations for ensemble docking were generated by clustering the conformations sampled in the ligand-free protein simulations (Table S1). These simulations were performed with the *Desmond-GPU version* (Schrödinger *Inc.*) and analysis for clustering was carried out by the Desmond Trajectory Clustering Tool in *Maestro*. We chose the clusters of receptor conformations based on the local dynamics of the ligand-binding site (local RMSD), in order to select the most relevant conformations and provide an affordable computational cost for ensemble docking. Clustering was based on heavy-atom RMSD of residues within 4 Å of the ligand-binding site. Only representative conformations of clusters with > 10 members were selected for ensemble docking. We had 2–6 clusters (Table S1) for each protein receptor in this work. Next, the top compounds from prescreening were docked to each of the cluster representative conformations in *Glide* (Schrödinger *Inc.*). The best Glide XP score^50^ and the corresponding pose from all the docking tests of these representative models are considered the ensemble docking score and pose respectively (Table S4). The stability of the complex models from ensemble docking was further evaluated by MD simulations for 120–250 ns with two trajectories for each complex.

Finally, selected complexes with one ligand were setup for cooperative docking (Table S5), which docked the top 10% of compounds from prescreening as the second ligand at the same binding site of each top complex model from ensemble docking by *Glide* (12 Å for the inner box; 15 Å for the outer box). For example, three clusters were identified for RdRp, and from each cluster a representative structure was used to provide the three structures for ensemble docking. The top complex models with saikosaponin I, macedonoside B, licoricesaponin F3 respectively were employed for cooperative docking (Table S5). Among all these complex models with two ligands bound, the best Glide XP score and the corresponding pose were reported as the cooperative docking results. To evaluate the stability and confirm the cooperativity, these complex models were simulated with two trajectories for 120–250 ns in *Desmond*, to further understand the potential cooperative binding. The simulations (i.e. RMSD, RMSF, and center of mass distance) were visualized and analyzed using *Maestro (version 2020-2)*, *VMD (version 1.9.3)*, and our in-house Python and Tcl scripts.

### System construction and simulation setup of molecular dynamics (MD)

We used MD simulations (i) to sample *apo* protein conformations for ensemble docking, and (ii) to validate complex structures from ensemble/cooperative docking. All the constructs were prepared in System Builder (Schrödinger *Inc.*) and relaxed using a multistage protocol which has been described in our previous work^51,52^. Parameters from the OPLS3e^53^ force field and the SPC water model^54^ were assigned.

Our production runs were performed in the NPT ensemble (300 K, 1 bar, Martyna–Tobias–Klein coupling scheme) with a time step of 2 fs in the *Desmond* MD engine. The particle mesh Ewald technique was used for the electrostatic calculations. Van der Waals and short-range electrostatics were cut off at 9.0 Å. The long-ranged electrostatics was updated every third simulation step. Each construct was simulated with two trajectories for 120–250 ns, and most of the systems showed consistent RMSF (Figure S4), which suggests sufficient sampling within our simulated length. Our total MD sampling reached 16 microseconds, with details provided in Tables S3–S5.

## Supplementary Information

Supplementary Information 1.

Supplementary Information 2.
