# Supplementary material for: A computational study of cooperative binding to multiple SARS-CoV-2 proteins
Source: Sci Rep. 2021 Aug 11;11:16307. doi: 10.1038/s41598-021-95826-6 (PMC8358031; doi:10.1038/s41598-021-95826-6)
Supplement: Supplementary file 2 — Supplementary Information 2. [file 41598_2021_95826_MOESM2_ESM.pdf]

Supporting Information

A Computational Study of Cooperative Binding to Multiple SARS-CoV-2 Proteins

Jianing Li,* Kyle T. McKay, Jacob M. Remington, Severin T. Schneebeli
Department of Chemistry, University of Vermont, Burlington, VT 05405

Corresponding Author: Jianing.li@uvm.edu

Table S1. Protein data set and summary of ensemble docking.

Proteins	PDBID	No. of clusters[a]	Box Center[b]	Notes
S protein (receptor binding domain)	7C8D	2	(102.0, 70.0, 72.7)	Chain B of the PDB structure
N protein (C-terminal dimerization domain)	6WJI	5	(7.6, -2.0, -14.4)	
3CLpro	7JYC	3	(123.5, 2.4, 22.4)	
PLpro	6WX4	5	(8.4, -29.1, -42.3)	
RdRp	7BV1	3	(83.7, 91.6, 109.3)	NSP12-NSP7-NSP8 complex
NSP3 (macro domain)	5RSO	3	(-48.6, -29.2, 4.8)	
Cat ACE2	7C8D	2	(99.5, 71.4, 78.3)	Chain A of the PDB structure
Human ACE2	6VW1	4	(91.0, 70.0, 65.0)[c]	

a. Clustering was based on heavy-atom RMSD of residues within 4 Å of the ligand or binding interface. Only the cluster size > 10 members (from the Desmond Trajectory Clustering Tool) was selected for ensemble docking.
b. Coordinates of the box center for alignments to the PDB structures.
c. Aligned to the cat ACE2.

Table S2. Summary of *Qingfei Paidu* decoction (QPD) ingredients.

QPD Ingredients	Latin Name	Chinese Name in Pinyin	No. of Compounds[a]	Ref.
Ephedra	*Ephedrae herba*	Ma Huang	52	1
Honey-fried licorice root	*Glycyrrhizae radix et rhiizoma*	Gan Cao	38	2-3
Bitter Almond	*Armeniacae semen amarum*	Xing Ren	12	4
Gypsum	*Gypsum fibrosum*	Shi Gao	0[b]	
Cassia twig	*Cinnamomi ramulus*	Gui Zhi	39	5
Rhizoma alismatis	*Alismatis rhizoma*	Ze Xie	19	6-7
Grifola	*Polyporus*	Zhu Ling	10	8
Atractylis ovata	*Atractylodis macrocephalae rhizoma*	Bai Zhu	21	9
Tuckahoe	*Poria*	Fu Ling	36	10
Chinese thorowax root	*Bupleuri radix*	Chai Hu	41	11
Scutellaria baicalensis	*Scutellariae radix*	Huang Qin	80	12-13
Rhizoma pinelliae	*Pinelliae rhizoma*	Jiang Ban Xia	13	14
Ginger	*Zingiberis rhizoma recens*	Sheng Jiang	93	15
Radix asteris	*Asteris radix et rhizoma*	Zi Wan	15	16
Fuyuka	*Farfarae flos*	Kuan Dong Hua	15	17
Belamcanda sinensis	*Belamcandae rhizoma*	Ye Gan	26	18-19
Asarum	*Asari radix et rhizoma*	Xi Xin	33	20
Chinese yam	*Dioscoreae rhizoma*	Shan Yao	17	21
Fructus aurantii immaturus	*Aurantii fructus immaturus*	Zhi Shi	27	22
Citri reticulatae pericarpium	*Citri reticulatae pericarpium*	Chen Pi	18	23
Agastache rugosa	*Pogostemonis herba*	Huo Xiang	20	24-25

a. These compounds in this work were selected from recent reviews and research articles listed in the references. Most small, volatile, toxic, and duplicate compounds were removed. Different protonation states (*p*H=7) and stereoisomers of each unique compounds were considered in the docking workflow.
b. Gypsum is a sulfate mineral composed of calcium sulfate dihydrate ($CaSO_4 \cdot 2H_2O$), which was not included.

Table S3. Summary of ligand-free simulations for clustering and selection of receptor structures in ensemble docking, totaling 2 μs. A 10-Å buffer distance was chosen to create the simulation box. NPT simulations at 300 K and 1 bar, simulation length = 250 ns, one replica. Protein Cα RMSD (to the crystal structure) of the last 20 ns is reported.

Protein Target	PDBID	No. of Residues	No. of Atoms	Box Size (Å^3)	Cα RMSD (Å)
S protein (receptor binding domain)	7C8D	195	32,319	56 × 70 × 83	1.7 ± 0.2
N protein (C-terminal dimerization domain)	6WJI	108	25,993	66 × 55 × 73	4.8 ± 0.1
3CLpro	7JYC	306	39,852	101 × 68 × 58	1.9 ± 0.2
PLpro	6WX4	320	59,426	111 × 67 × 81	2.3 ± 0.2
RdRp	7BV1	902	118,436	107 × 131 × 86	3.1 ± 0.2
NSP3 (macro domain)	5RSO	167	24,517	62 × 60 × 66	1.4 ± 0.1
Cat ACE2	7C8D	597	78,656	101 × 86 × 92	2.1 ± 0.3
Human ACE2	6VW1	596	66,181	80 × 98 × 84	1.8 ± 0.2

Table S4. Summary of ligand-bound simulations to confirm the complex stability, totaling 7 μs. Each complex structures was taken from the best pose from ensemble docking. A 10- or 15-Å buffer distance was chosen to create the simulation box. NPT simulations at 300 K and 1 bar, two replicas (250 and 120 ns respectively). Protein Cα RMSD (to the crystal structure) and local ligand RMSD (to the initial/final frame, protein alignment, heavy atom) of the last 20 ns is reported.

Protein Target	Ligand	No. of Atoms	Box Size (Å^3)	XP Score (kcal/mol)	Protein Cα RMSD (Å)	Ligand RMSD (Å)	Note[a]
S protein	rutin	46,906	64 × 76 × 92	-7.2	2.0 ± 0.1	4.7 ± 0.9[b]<br>2.3 ± 1.1[c]	
	chrysin 7-o-β-gentiobioside	30,728	57 × 66 × 82[d]	-6.3	2.1 ± 0.1	5.6 ± 1.0[b]<br>2.5 ± 1.3[c]	
	narcissin	30,721	57 × 66 × 82[d]	-5.7	1.9 ± 0.1	12.7 ± 2.7[b]<br>4.9 ± 2.8[c]	
	tectorigenin 7-o-gentiobioside	30,652	64 × 59 × 82[d]	-6.2	2.0 ± 0.2	11.6 ± 1.0[b]<br>2.5 ± 1.4[c]	
	ephedrine	27,779	53 × 67 × 80[d]	4.9	2.3 ± 0.2	4.4 ± 0.1[b]<br>2.6 ± 0.4[c]	
N protein	rutin	31,406	77 × 62 × 66	-8.0	5.1 ± 0.2	5.7 ± 0.5[b]<br>2.2 ± 0.8[c]	
	ephedrine	21,591	67 × 51 × 63[d]	-5.3	6.1 ± 0.6		Dissociation after 6 ns
3CLpro	rutin	55,540	105 × 78 × 68	-12.2	1.8 ± 0.1	7.6 ± 0.3[b]<br>1.6 ± 0.6[c]	$d_{Rutin-C145}$ = 9.2 ± 0.4 Å
	ephedrine	34,944	98 × 65 × 55[d]	-5.1	2.1 ± 0.1	5.8 ± 0.3[b]<br>2.0 ± 0.5[c]	
PLpro	rutin	49,936	101 × 64 × 77[d]	-7.8	2.5 ± 0.3	6.3 ± 1.0[b]<br>4.5 ± 2.7[c]	$d_{Rutin-C111}$ = 10.7 ± 0.9 Å
RdRp	rutin	143,616	114 × 141 × 90	-9.3	3.5 ± 0.1	5.9 ± 0.5[b]<br>1.3 ± 0.6[c]	
	saikosaponin I	108,145	104 × 131 × 80[d]	-11.8	2.8 ± 0.1	5.3 ± 0.2[b]<br>1.8 ± 0.4[c]	Dissociation after 48 ns in another replica
	macedonoside B	99,432	104 × 120 × 80[d]	-11.3	3.0 ± 0.1	4.0 ± 0.6[b]<br>2.1 ± 0.7[c]	Dissociation after 48 ns in another replica
	licoricesaponin F3	104,891	105 × 128 × 80[d]	-10.4	2.9 ± 0.2	4.3 ± 0.4[b]<br>3.0 ± 0.9[c]	
	ephedrine	108,336	103 × 128 × 82[d]	-4.1	2.7 ± 0.2	7.8 ± 1.3[b]<br>4.3 ± 1.0[c]	60 ns in length
NSP3	rutin	39,349	71 × 77 × 73	-10.5	1.2 ± 0.1	1.6 ± 0.3[b]<br>2.8 ± 0.8[c]	
	ephedrine	20,381	59 × 66 × 62[d]	-2.8	1.3 ± 0.2		Dissociation after 8 ns
Cat ACE2	rutin	94,902	109 × 90 × 98	-7.3	2.1 ± 0.2	6.8 ± 0.5[b]<br>2.9 ± 1.3[c]	
	vicenin2	67,340	90 × 89 × 85[d]	-6.3	3.6 ± 0.2	6.5 ± 0.8[b]<br>1.9 ± 0.9[c]	

	ephedrine	68,762	96 × 82 × 88[d]	-4.4	1.9 ± 0.1		Dissociation after 4 ns
Human ACE2	rutin	66,181	80 × 98 × 84[d]	-5.1	2.4 ± 0.2	9.7 ± 0.9[b] 4.1 ± 2.9[c]	
	narcissin	66,199	81 × 98 × 84[d]	-6.1	2.4 ± 0.2	8.4 ± 0.6[b] 2.9 ± 1.4[c]	

a. The distance was measured as the distance between the heavy-atom center of rutin and the cysteine S atom.
b. The initial structure from the docking pose as the reference.
c. The final snapshot as the reference.
d. 10-Å buffer.

Table S5. Summary of cooperative binding simulations, totaling 8 μs. Each complex structures with two compounds bound was obtained from the best pose from cooperative docking. A 10-Å buffer distance was chosen to create the simulation box. NPT simulations at 300 K and 1 bar, two replicas (250 and 120 ns respectively). Protein Cα RMSD (to the crystal structure) and local ligand RMSD (to the final frame, protein alignment, heavy atom) of the last 20 ns is reported.

**Protein Target**	**Protein Cα RMSD (Å)**		**Ligand-1[a]**			**Ligand-2[b]**		**COM Distance (Å)**
		**Ligand Name**	**Glide XP Score (kcal/mol)**	**RMSD (Å)**	**Ligand Name**	**Glide XP Score (kcal/mol)**	**RMSD (Å)**	
S protein	1.7 ± 0.2	rutin	-7.2	4.5 ± 0.5 1.7 ± 0.6	rutin	-7.7	6.2 ± 0.8 3.8 ± 1.4	6.7 ± 0.7
	2.3 ± 0.1	rutin	-7.2	5.2 ± 0.5 2.2 ± 0.7	coumarin glycoside	-5.0	12.0 ± 0.9 4.0 ± 1.4	7.8 ± 0.8
	2.0 ± 0.1	rutin	-7.2	4.5 ± 0.8 2.2 ± 0.9	narcissin	-6.0	7.0 ± 1.9 3.5 ± 2.8	7.9 ± 1.3
	2.4 ± 0.2	rutin	-7.2	6.8 ± 0.7 2.9 ± 1.0	tectorigenin 7-O-gentiobioside	-7.0	12.7 ± 0.6 4.0 ± 1.4	4.5 ± 0.3
	2.2 ± 0.2	rutin	-7.2	10.0 ± 2.5 8.0 ± 3.3	chrysin 7-o- β-gentiobioside	-7.7	13.0 ± 1.2 2.9 ± 1.1	8.8 ± 1.8
	2.2 ± 0.1	rutin	-7.2	16.9 ± 2.1 5.2 ± 3.5	kaempferol 3-O-neohesperidoside	-8.3	10.7 ± 1.9 3.7 ± 1.2	20.0 ± 1.9
3CLpro	1.9 ± 0.2	rutin	-12.2	8.0 ± 0.8 6.3 ± 2.4	rutin	-9.5	9.2 ± 0.6 3.8 ± 1.2	5.9 ± 0.4
	1.8 ± 0.1	rutin	-12.2	6.2 ± 0.3 0.9 ± 0.3	lucenin3	-10.4	11.8 ± 0.3 1.1 ± 0.3	10.6 ± 0.3
	1.7 ± 0.2	lucenin3	-12.4	1.2 ± 0.2 0.8 ± 0.3	rutin	-8.7	11.6 ± 0.5 1.5 ± 0.7	7.6 ± 0.3
PLpro	2.2 ± 0.2	rutin	-7.8	12.3 ± 0.6 3.7 ± 1.9	rutin	-5.6	9.0 ± 0.4 2.6 ± 0.9	6.2 ± 0.6
	2.4 ± 0.3	rutin	-7.8	11.6 ± 1.2 3.3 ± 1.6	narcissin	-5.4	12.5 ± 1.6 4.5 ± 2.1	8.3 ± 0.5
RdRp	4.2 ± 0.1	rutin	-9.3	6.1± 0.2 0.7 ± 0.3	rutin	-7.1	8.4 ± 0.4 1.4 ± 0.5	6.8 ± 0.3
	3.8 ± 0.1	rutin	-9.3	1.9 ±0.4 1.0 ± 0.3	tectorigenin 7-O-xylosylglucoside	-11.8	2.5 ± 0.3 1.1 ± 0.3	6.8 ± 0.2
	3.1 ± 0.1	saikosaponin I	-11.8	3.0 ± 0.1 1.6 ± 0.1	rutin	-10.7	2.6 ± 0.2 0.8 ± 0.3	5.8 ± 0.2
	3.2 ± 0.1	macedonoside B	-11.3	2.2 ± 0.3 1.2 ± 0.3	rutin	-11.2	2.4 ± 0.5 2.0 ± 0.2	7.2 ± 0.3
	3.7 ± 0.1	licoricesaponin F3	-10.4	4.3 ± 0.4 1.4 ± 0.3	rutin	-10.9	6.4 ± 0.3 1.8 ± 0.5	6.5 ± 0.3
Cat ACE2	2.1 ± 0.1	rutin	-7.3	14.6 ± 0.3 0.9 ± 0.3	rutin	-4.4	22.4 ± 0.6 3.0 ± 1.1	7.1 ± 0.5
	2.4 ± 0.2	rutin	-7.3	17.8 ± 0.5 2.9 ± 1.7	chrysin 7-o- β-gentiobioside	-5.5	20.1 ± 0.8 5.1 ± 1.9	6.9 ± 0.4
Human ACE2	2.1 ± 0.2	rutin	-5.1	8.4 ± 1.0 3.5 ± 1.5	rutin	-7.2	18.3 ± 1.1 3.1 ± 1.0	24.0 ± 0.8

	2.3 ± 0.3	rutin	-5.1	13.4 ± 1.2<br>9.5 ± 3.7	chrysin 7-o- β-gentiobioside	-4.2	18.2 ± 1.3<br>6.1 ± 2.0	6.5 ± 0.7
	2.4 ± 0.3	narcissin	-6.1	9.1 ± 1.9<br>7.6 ± 2.8	rutin	-4.6	12.1 ± 2.8<br>8.9 ± 3.0	6.6 ± 1.1

a. Docking to the ligand-free protein. Same as in Table S4, the first RMSD was compared with the initial model from docking; the second RMSD was compared with the final snapshot of the MD simulation.
b. Docking to the ligand-1-bound protein. Same as in Table S4, the first RMSD was compared with the initial model from docking; the second RMSD was compared with the final snapshot of the MD simulation.

Table S6. Drug-likeness properties of selected natural compounds predicted by SwissADME (http://www.swissadme.ch).

Compound	MW (g/mol)	Num. heavy atoms	Num. rotatable bonds	Num. HB acceptors	Num. HB donors	Log Po/w	Water Solubility	P450 inhibition	PAINS alert
**Amygdalin**	457	32	7	12	7	-2.32	Soluble	No	0
**Rutin**	611	43	6	16	10	-1.12	Soluble	No	1
**Narcissin**	625	44	7	16	9	-0.80	Soluble	No	0
**Hyperin**	464	33	4	12	8	-0.15	Soluble	No	1
**Hyperin 5-O-galactopyranoside**	627	44	7	17	11	-2.61	Soluble	No	1
**Hyperin 7-O-D-xylopyranoside**	597	42	6	16	10	-1.77	Soluble	No	1
**Hyperin 6’’-gallate**	617	44	7	16	10	-0.11	Soluble	No	1
**Tectorigenin 7-O-gentiobioside**	625	44	8	16	9	-1.46	Soluble	No	0
**Kaempferol 7-O-neohesperidoside**	595	42	6	15	9	-0.97	Soluble	No	0

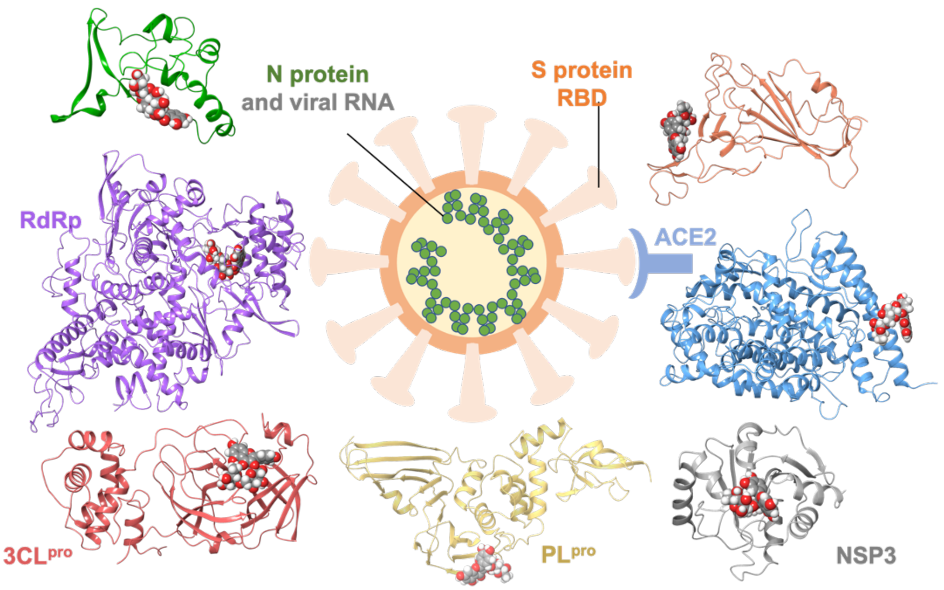

Figure S1. Cartoon illustration of the SARS-CoV-2-related proteins in this work. The best poses from docking one rutin molecule to the protein structures (see PDBIDs in Table S1) are shown. The protein structures are represented by cartoon while atoms of rutin are shown as spheres (carbon: grey; oxygen: red; hydrogen: white).

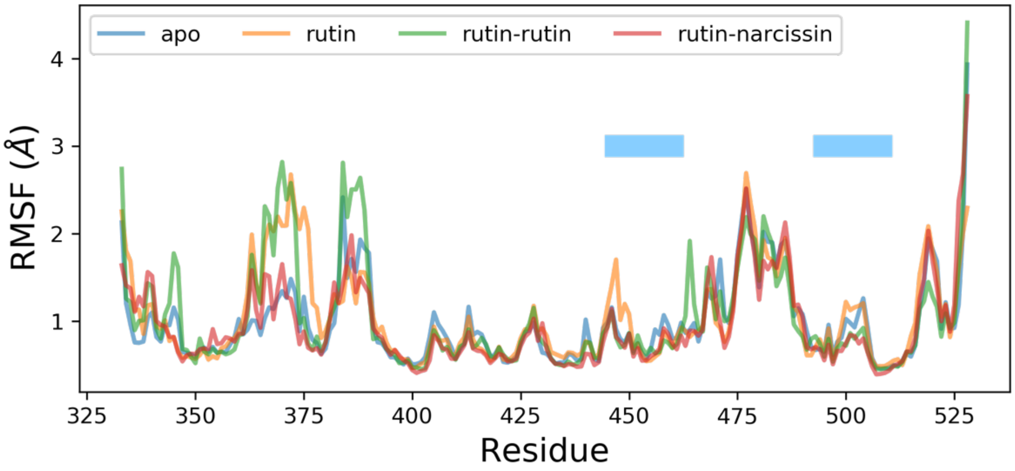

Figure S2. Root-mean-square fluctuations (RMSFs) of four S protein RBD simulations. The β1' (residues 446–458) and β2' (residues 490–502) regions are highlighted with light blue boxes. In direct contact with the bound compounds, the β2' dynamics is reduced by cooperative compound binding.

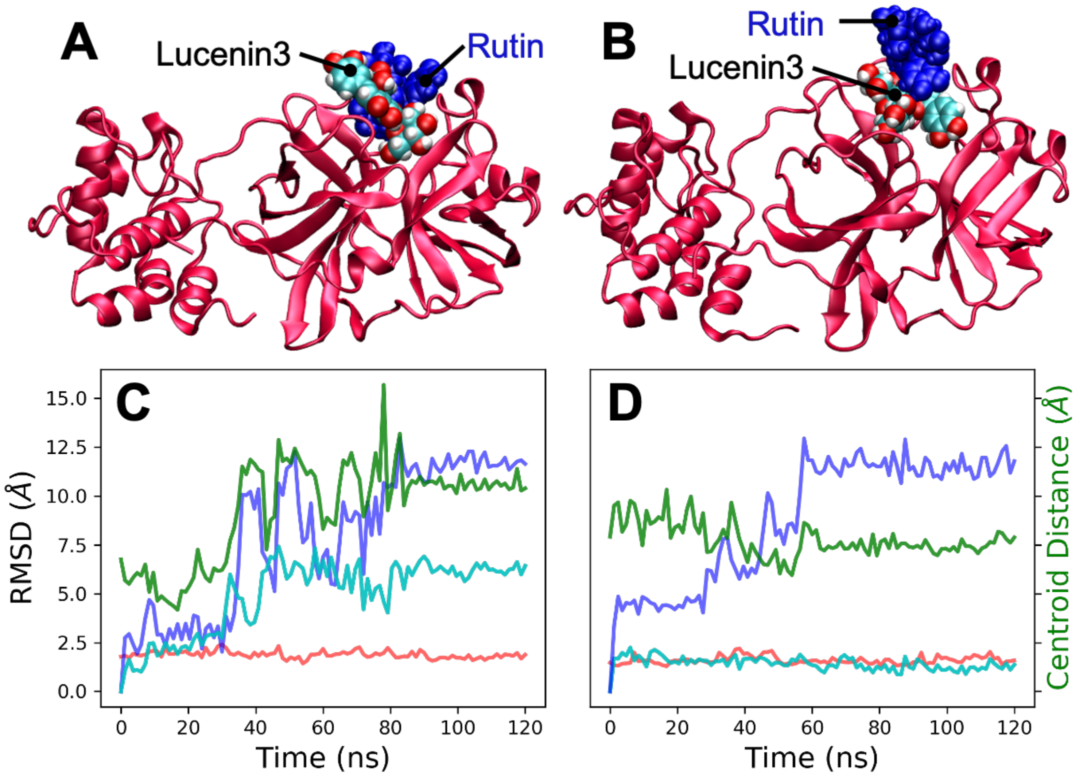

Figure S3. Cartoon illustration of SARS-CoV-2 3CL^{pro} in complex with **(A)** rutin and lucenin3 and **(B)** lucenin3 and rutin, extracted from the final snapshots of 120-ns MD simulations. **(C-D)** Time evolution of protein/ligand RMSDs and the centroid distance of the two ligands. (protein backbone: red; rutin: blue; lucenin3: cyan; centroid distance: green). This is an example to depict the different binding poses from cooperative binding: the complex with lucenin3 as the first ligand in (B & D) appeared to be more stable than the complex with rutin as the first ligand in (A & C).

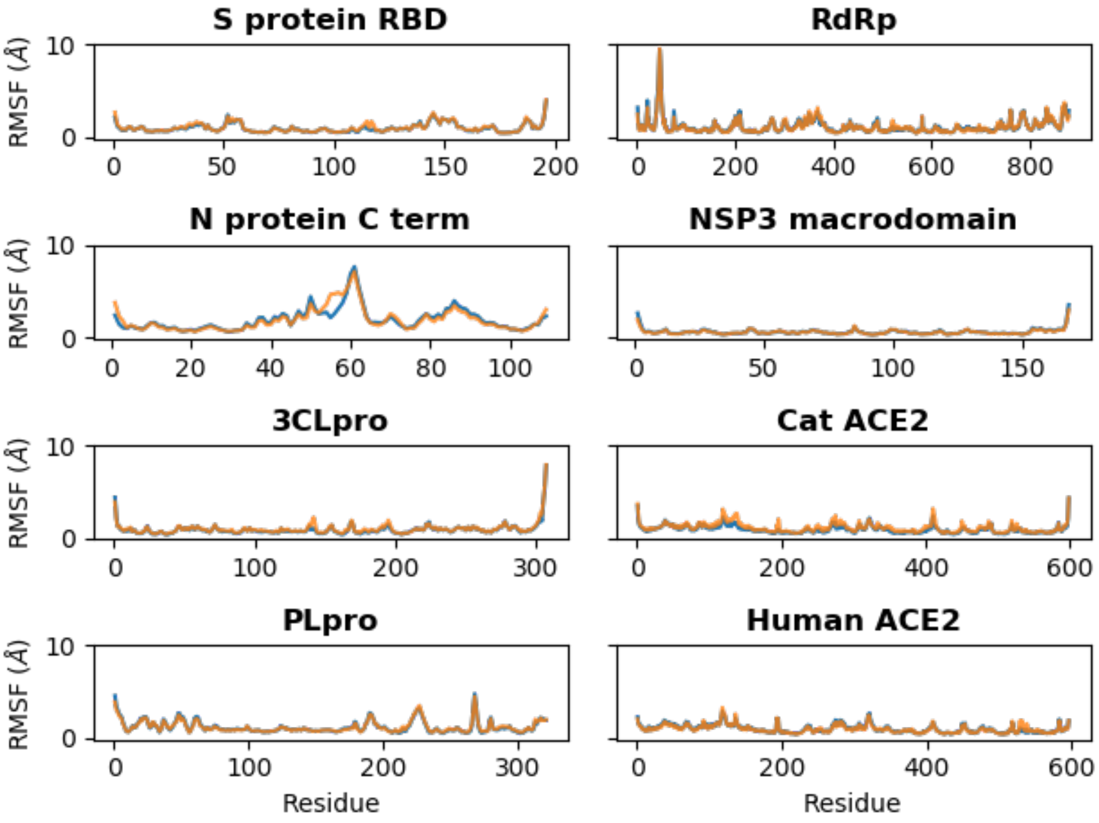

Figure S4. Comparison of protein backbone RMSF of the 120- (blue) and 250-ns (orange) simulations. The overlap of the RMSF plots for all the proteins indicate sufficient sampling with a 120-ns simulation length.

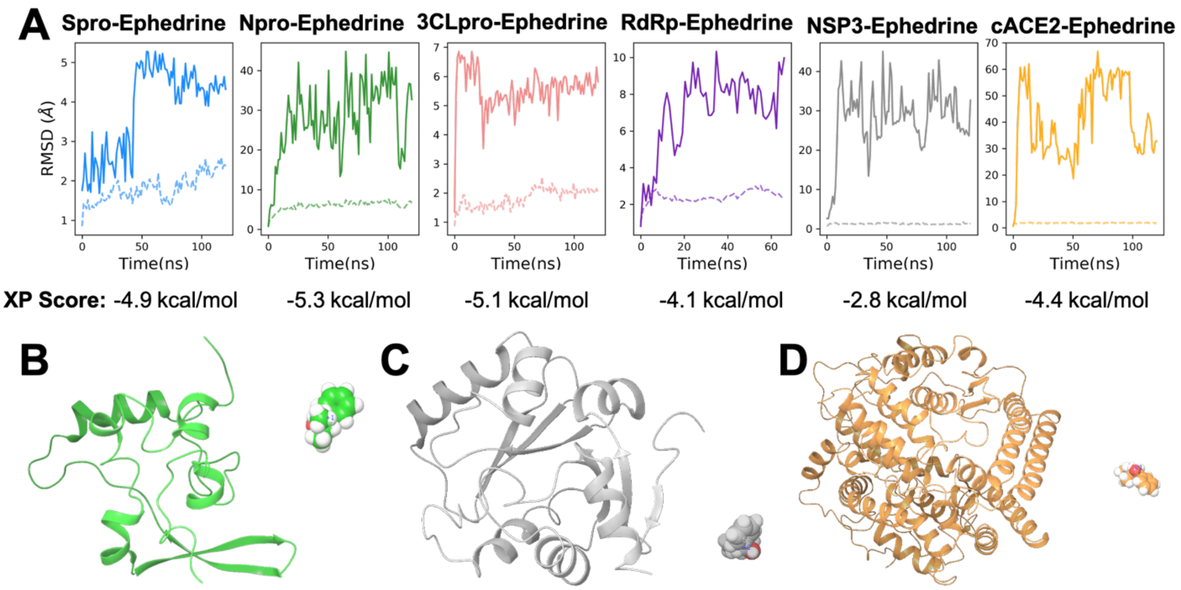

Figure S5. Ephedrine (from the Ephedra herb) was used as a negative control compound, in comparison with Rutin (shown in Figure 2). In our docking studies, the Glide XP scores of ephedrine were generally low (between -2 to -6 kcal/mol). **(A)** Time evolution of protein (dash line) and ligand (solid line) RMSD, with the complex aligned to the docking poses. **(B-D)** Final snapshots (at 60 or 120 ns) show clear dissociation of the Npro-Ephedrine (green), NSP3-Ephedrine (grey), and cat ACE2-Ephedrine (orange) complexes.

References:

1. González-Juárez, D. E.; Escobedo-Moratilla, A.; Flores, J.; Hidalgo-Figueroa, S.; Martínez-Tagüeña, N.; Morales-Jiménez, J.; Muñiz-Ramírez, A.; Pastor-Palacios, G.; Pérez-Miranda, S.; Ramírez-Hernández, A.; Trujillo, J.; Bautista, E., A Review of the Ephedra genus: Distribution, Ecology, Ethnobotany, Phytochemistry and Pharmacological Properties. *Molecules* **2020,** *25* (14), 3283.

2. Pastorino, G.; Cornara, L.; Soares, S.; Rodrigues, F.; Oliveira, M. B. P. P., Liquorice (Glycyrrhiza glabra): A phytochemical and pharmacological review. *Phytotherapy Research* **2018,** *32* (12), 2323-2339.

3. Li, F.; Liu, B.; Li, T.; Wu, Q.; Xu, Z.; Gu, Y.; Li, W.; Wang, P.; Ma, T.; Lei, H., Review of Constituents and Biological Activities of Triterpene Saponins from Glycyrrhizae Radix et Rhizoma and Its Solubilization Characteristics. *Molecules* **2020,** *25* (17), 3904.

4. Gao, K.; Song, Y.-P.; Song, A., Exploring active ingredients and function mechanisms of Ephedra-bitter almond for prevention and treatment of Corona virus disease 2019 (COVID-19) based on network pharmacology. *BioData Mining* **2020,** *13* (1), 19.

5. Liu, J.; Zhang, Q.; Li, R.-L.; Wei, S.-J.; Huang, C.-Y.; Gao, Y.-X.; Pu, X.-F., The traditional uses, phytochemistry, pharmacology and toxicology of Cinnamomi ramulus: a review. *Journal of Pharmacy and Pharmacology* **2020,** *72* (3), 319-342.

6. Zhao, W.; Huang, X.; Li, X.; Zhang, F.; Chen, S.; Ye, M.; Huang, M.; Xu, W.; Wu, S., Qualitative and Quantitative Analysis of Major Triterpenoids in Alismatis Rhizoma by High Performance Liquid Chromatography/Diode-Array Detector/Quadrupole-Time-of-Flight Mass Spectrometry and Ultra-Performance Liquid Chromatography/Triple Quadrupole Mass Spectrometry. *Molecules* **2015,** *20* (8), 13958-13981.

7. Zhang, L.-L.; Xu, W.; Xu, Y.-L.; Chen, X.; Huang, M.; Lu, J.-J., Therapeutic potential of Rhizoma Alismatis: a review on ethnomedicinal application, phytochemistry, pharmacology, and toxicology. *Annals of the New York Academy of Sciences* **2017,** *1401* (1), 90-101.

8. Zjawiony, J. K., Biologically Active Compounds from Aphyllophorales (Polypore) Fungi. *Journal of Natural Products* **2004,** *67* (2), 300-310.

9. Zhang, W.-J.; Zhao, Z.-Y.; Chang, L.-K.; Cao, Y.; Wang, S.; Kang, C.-Z.; Wang, H.-Y.; Zhou, L.; Huang, L.-Q.; Guo, L.-P., Atractylodis Rhizoma: A review of its traditional uses, phytochemistry, pharmacology, toxicology and quality control. *J Ethnopharmacol* **2021,** *266*, 113415-113415.

10. Ríos, J. L., Chemical constituents and pharmacological properties of Poria cocos. *Planta Medica* **2011,** *77* (7), 681-91.

11. Yang, F.; Dong, X.; Yin, X.; Wang, W.; You, L.; Ni, J., Radix Bupleuri: A Review of Traditional Uses, Botany, Phytochemistry, Pharmacology, and Toxicology. *Biomed Res Int* **2017,** *2017*, 7597596-7597596.

12. Li, C.; Lin, G.; Zuo, Z., Pharmacological effects and pharmacokinetics properties of Radix Scutellariae and its bioactive flavones. *Biopharmaceutics & Drug Disposition* **2011,** *32* (8), 427-445.

13. Wang, Z.-L.; Wang, S.; Kuang, Y.; Hu, Z.-M.; Qiao, X.; Ye, M., A comprehensive review on phytochemistry, pharmacology, and flavonoid biosynthesis of Scutellaria baicalensis. *Pharm Biol* **2018,** *56* (1), 465-484.

14. Su, T.; Tan, Y.; Tsui, M.-S.; Yi, H.; Fu, X.-Q.; Li, T.; Chan, C. L.; Guo, H.; Li, Y.-X.; Zhu, P.-L.; Tse, A. K. W.; Cao, H.; Lu, A.-P.; Yu, Z.-L., Metabolomics reveals the mechanisms for the cardiotoxicity of Pinelliae Rhizoma and the toxicity-reducing effect of processing. *Scientific Reports* **2016,** *6* (1), 34692.

15. Zhang, M.; Zhao, R.; Wang, D.; Wang, L.; Zhang, Q.; Wei, S.; Lu, F.; Peng, W.; Wu, C., Ginger (Zingiber officinale Rosc.) and its bioactive components are potential resources for health beneficial agents. *Phytotherapy Research* **2021,** *35* (2), 711-742.

16. Tang, J.; Cheng, M.; Hattori, M., Pyrrolizidine alkaloid profile in a traditional Chinese herbal medicine Chuan Zi Wan (Ligulariae Radix et Rhizoma) by liquid chromatography/electrospray ionization ion trap mass spectrometry. *Analytical Methods* **2012,** *4* (9), 2797-2808.

17. Kim, M. R.; Lee, J. Y.; Lee, H. H.; Aryal, D. K.; Kim, Y. G.; Kim, S. K.; Woo, E. R.; Kang, K. W., Antioxidative effects of quercetin-glyco sides isolated from the flower buds of Tussilago farfara L. *Food and Chemical Toxicology* **2006,** *44* (8), 1299-1307.

18. Wozniak, D.; Janda, B.; Kapusta, I.; Oleszek, W.; Matkowski, A., Antimutagenic and anti-oxidant activities of isoflavonoids from Belamcanda chinensis (L.) DC. *Mutation Research-Genetic Toxicology and Environmental Mutagenesis* **2010,** *696* (2), 148-153.

19. Kang, S. W.; Kim, M. C.; Kim, C. Y.; Jung, S. H.; Um, B. H., The rapid identification of isoflavonoids from Belamcanda chinensis by LC-NMR and LG-MS. *Chemical & Pharmaceutical Bulletin* **2008,** *56* (10), 1452-1454.

20. Michl, J.; Bello, O.; Kite, G. C.; Simmonds, M. S. J.; Heinrich, M., Medicinally Used Asarum Species: High-Resolution LC-MS Analysis of Aristolochic Acid Analogs and In vitro Toxicity Screening in HK-2 Cells. *Front Pharmacol* **2017,** *8*, 215-215.

21. Epping, J.; Laibach, N., An underutilized orphan tuber crop—Chinese yam : a review. *Planta* **2020,** *252* (4), 58.

22. Wu, J.; Huang, G.; Li, Y.; Li, X., Flavonoids from Aurantii Fructus Immaturus and Aurantii Fructus: promising phytomedicines for the treatment of liver diseases. *Chinese Medicine* **2020,** *15* (1), 89.

23. Favela-Hernández, J. M. J.; González-Santiago, O.; Ramírez-Cabrera, M. A.; Esquivel-Ferriño, P. C.; Camacho-Corona, M. d. R., Chemistry and Pharmacology of Citrus sinensis. *Molecules* **2016,** *21* (2), 247-247.

24. Cao, P.; Xie, P.; Wang, X.; Wang, J.; Wei, J.; Kang, W.-y., Chemical constituents and coagulation activity of Agastache rugosa. *BMC Complementary and Alternative Medicine* **2017,** *17* (1), 93.

25. Zielińska, S.; Matkowski, A., Phytochemistry and bioactivity of aromatic and medicinal plants from the genus Agastache (Lamiaceae). *Phytochem Rev* **2014,** *13* (2), 391-416.
